# Exploration of Convective and Infrared Drying Effect on Image Texture Parameters of ‘Mejhoul’ and ‘Boufeggous’ Date Palm Fruit Using Machine Learning Models

**DOI:** 10.3390/foods13111602

**Published:** 2024-05-21

**Authors:** Younes Noutfia, Ewa Ropelewska

**Affiliations:** Fruit and Vegetable Storage and Processing Department, The National Institute of Horticultural Research, Konstytucji 3 Maja 1/3, 96-100 Skierniewice, Poland; younes.noutfia@inhort.pl

**Keywords:** *Phoenix dactylifera* L., image features, flatbed scanner, artificial intelligence, classification models

## Abstract

Date palm (*Phoenix dactylifera* L.) fruit samples belonging to the ‘Mejhoul’ and ‘Boufeggous’ cultivars were harvested at the Tamar stage and used in our experiments. Before scanning, date samples were dried using convective drying at 60 °C and infrared drying at 60 °C with a frequency of 50 Hz, and then they were scanned. The scanning trials were performed for two hundred date palm fruit in fresh, convective-dried, and infrared-dried forms of each cultivar using a flatbed scanner. The image-texture parameters of date fruit were extracted from images converted to individual color channels in RGB, Lab, XYZ, and UVS color models. The models to classify fresh and dried samples were developed based on selected image textures using machine learning algorithms belonging to the groups of Bayes, Trees, Lazy, Functions, and Meta. For both the ‘Mejhoul’ and ‘Boufeggous’ cultivars, models built using Random Forest from the group of Trees turned out to be accurate and successful. The average classification accuracy for fresh, convective-dried, and infrared-dried ‘Mejhoul’ reached 99.33%, whereas fresh, convective-dried, and infrared-dried samples of ‘Boufeggous’ were distinguished with an average accuracy of 94.33%. In the case of both cultivars and each model, the higher correctness of discrimination was between fresh and infrared-dried samples, whereas the highest number of misclassified cases occurred between fresh and convective-dried fruit. Thus, the developed procedure may be considered an innovative approach to the non-destructive assessment of drying impact on the external quality characteristics of date palm fruit.

## 1. Introduction

Date palm (*Phoenix dactylifera* L.) is a very important fruit crop grown in many regions of the world, especially in hot and dry areas, and it is considered a promising tree for the irrigated dry zones of developing nations [1,2]. For many nations, date fruit is an essential subsistence food [1] and an important component of a healthy diet due to its functional properties as well as its high sugar content, flavonoids, anthocyanins, phenols, vitamins, minerals, and carotenoids [3]. Hence, the nutritional composition of date fruit differs according to several factors and can range from 44 to 88% for total sugar, 2.3–5.6% for protein, 6.4–11.5% for fibers, 0.2–0.5% for fat, and 0.2–0.5% for oil. Thus, both edible parts of date fruit and seeds can be used in food products as a rich source of biochemical and functional compounds such as antioxidants and fibers [1], especially at the Tamar stage. 

At this complete stage of maturity, the fruit usually has a reduced moisture content of less than 20–25%, and it is considered appropriate for consumption in many regions and for storage under specific and mastered conditions [4,5]. However, some physiological and physical disorders can occur at this stage, mainly related to skin separation and sugar spots. These visual defects negatively impact the visual quality of the fruit and its market value [1]. Therefore, drying can provide shelf-life extension [6] and postpone the perishability and degeneration of date fruit.

Drying as a pretreatment, in association with efficient storage availability, ensures high-quality date fruit all year round in markets and in places where they are not produced [7]. By removing water, transport efficiency is increased by reducing the product volume as well as packaging costs. Besides this logistical improvement, moisture level, fruit sugars, and other biochemical compounds are more concentrated, allowing for more protection against deterioration factors, microorganism proliferation and growth, undesired chemical reactions, and high risk of deterioration during storage [8], leading to preservation of quality properties and shelf life extension. Moreover, date fruit may be dried using various methods, such as open sun drying, solar drying, hot air oven drying, vacuum drying, microwave drying, microwave vacuum drying, drum drying, or freeze drying [9].

The sun drying of date fruit has been the most common way throughout history. However, the process is challenging because of the inability to control drying parameters that may fluctuate drastically depending on climatic conditions. Furthermore, the sun drying process requires a long time while date fruit is under uncontrolled conditions of illumination and airflow, in addition to a possible source of contamination with insects, sand particles, soil, and dust. Thus, sun drying is not considered an effective method to apply for date fruit in the perspective of quality preservation and amelioration prior to further postharvest operations (e.g., storage). Consequently, other drying methods are more suitable for maintaining and improving the internal and external qualities of date fruit: homogenous skin color; adequate skin hardness; and high preservation of nutrient compounds [10,11,12,13]. One of the most common and effective methods is convective drying, which ensures a reduction in moisture content and quality preservation [14]. Also, infrared drying can be more beneficial compared with conventional drying due to rapid processing, shorter heating times, chemical compound preservation, and reduced risk of flavor loss [15]. Artificial intelligence and image analysis can be considered innovative and alternative approaches to assess the quality of date fruit before and after drying instead of tedious, time-consuming, and destructive analyses [5]. In doing so, machine vision enables the quantitative analysis of the quality of the qualitative criteria of the sample. Machine learning (ML) models can be successful in the classification of different samples of date fruit based on selected image parameters using several algorithms and models (e.g., traditional ML algorithms) [16]. Furthermore, image textures and determined geometric parameters using image analysis can be useful in the objective characterization of date fruit [17].

Considering the previous elements, the objective of this study was to compare the effect of convective and infrared drying on the quality of ‘Mejhoul’ and ‘Boufeggous’ date palm fruit in terms of external appearance. The assessment of the fruit quality was performed objectively and non-destructively using innovative artificial intelligence models developed based on selected image texture parameters.

## 2. Materials and Methods

### 2.1. Materials

Two well-known commercial cultivars of Moroccan date palm fruit, ‘Mejhoul’ and ‘Boufeggous’, were used in these experiments. Fruit samples were harvested at the Tamar stage in November 2023 in an orchard located at Henabou-Erfoud in southeastern Morocco (31°26′10″ N, 4°13′58″ W) and stored in cardboard boxes in a cold room at 2–4 °C until experiment starting. Before and after drying experiments, two hundred fruits of ‘Mejhoul’ and ‘Boufeggous’ without any visual defects were subjected to imaging.

### 2.2. Date Fruit Drying

Date fruit belonging to ‘Mejhoul’ and ‘Boufeggous’ cultivars were dried using a CONVECO semi-industrial dryer (CONVECO Sp. z o.o., Glinianka, Poland) equipped with a power supply cabinet, control cabinet, heat recovery (recuperation) units, and a ventilation duct system. For both convective and infrared drying, two technological repetitions were carried out, and the same temperature of 60 °C was used. For the infrared drying, the waving range was at a frequency of 10 min at a radiation distance of 170 mm with a radiation power of 50 Hz.

#### 2.2.1. Convective Drying

**Figure 1:**
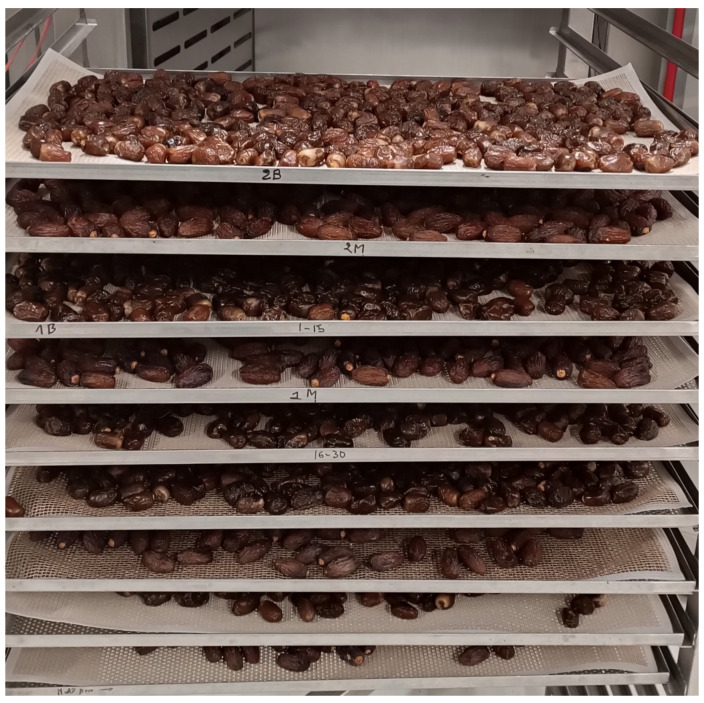
Illustration of convective drying of ‘Mejhoul’ and ‘Boufeggous’.

For each technological repetition, date fruit samples were spread out in one layer into trays placed at different levels of the drying chamber rack (Figure 1). Each tray contained approximately 3–4 kg of ‘Mejhoul’ or ‘Boufeggous’ to provide a total amount of dried material of approximately 25–26 kg per cultivar and per technological repetition. This process was performed through hot air at a temperature of 60 °C, and the airflow side (right or left) was changed every 10 min. This process was carried out until reaching the desired weight loss and estimated water content (achieved in 240 min).

#### 2.2.2. Infrared Drying

**Figure 2:**
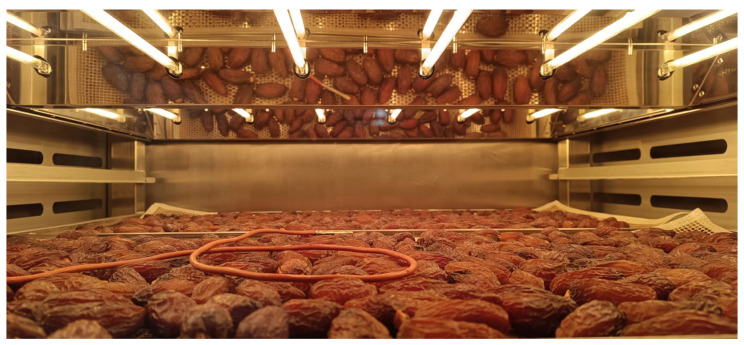
Illustration of infrared drying of ‘Mejhoul’.

The surfaces of the date samples were heated using lamps (radiant heaters) located above trays with date fruit, with a distance (between lamps and fruit samples) of 17 cm (Figure 2). The infrared drying was performed at parameters of 60 °C and 50 Hz. The whole process lasted 150 min, and the date fruit was side-changed in the middle of the process so that the lower side of the fruit was on top and heated consequently by the lamps. The changing side of airflow (right or left) was every 10 min. As for convective drying, infrared drying was performed in two technological repetitions, and the airflow side (right or left) was changed every 10 min.

### 2.3. Image Analysis

**Figure 3:**
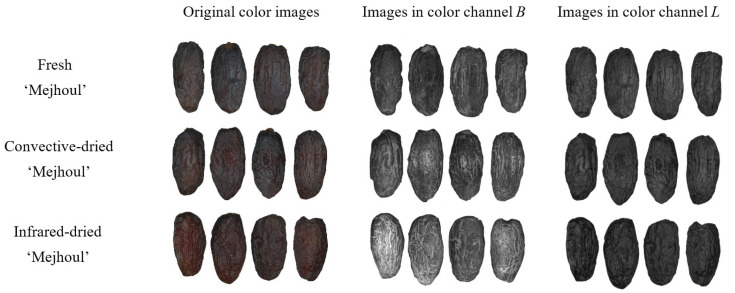
Exemplary original color images and images in selected color channels of fresh, convective-dried (CD), and infrared-dried (ID) date fruit ‘Mejhoul’. *B* refers to blue, and *L* refers to lightness.

**Figure 4:**
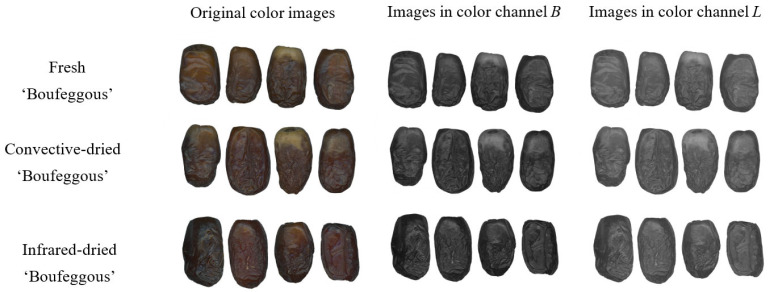
Exemplary original color images and images in selected color channels of fresh, convective-dried, and infrared-dried date fruit ‘Boufeggous’. *B* refers to blue, and *L* refers to lightness.

The fresh, convective-dried, and infrared-dried date fruit were scanned using the Epson Perfection flatbed scanner (Epson, Suwa, Nagano, Japan) on a white background covered by a box and saved in TIFF format. Twenty fruits were in one image, and the scanning was performed for two hundred date palm fruit samples of ‘Mejhoul’ and ‘Boufeggous’ in fresh, convective-dried, and infrared-dried forms. Before image processing, the white background was changed to black to facilitate image segmentation and separation of fruit from the background, and images were saved in BMP format. Image processing, including image segmentation, ROI (region of interest) determination, and texture extraction, was performed using MaZda 4.7 software (Łódź University of Technology, Institute of Electronics, Łódź, Poland) [18,19,20]. The date fruit images were converted to individual color channels *R*, *G*, *B*, *L*, *a*, *b*, *X*, *Y*, *Z*, *U*, *V*, and *S*. Exemplary original color images and images in selected color channels of fresh, convective-dried, and infrared-dried date fruit of ‘Mejhoul’ and ‘Boufeggous’ are presented respectively in Figure 3 and Figure 4. The dried fruit samples were of better quality in terms of appearance and characterized by shinier skin, especially in the case of infrared-dried fruit. The image segmentation was performed based on the brightness threshold, and the lighter date fruit samples were separated from the black background. Each whole date fruit was treated as an ROI, and for each ROI, 2160 image textures were computed based on the run-length matrix, co-occurrence matrix, histogram, gradient map, and autoregressive model.

### 2.4. Mean Comparison of Selected Image Textures

Graphs of mean values of selected image textures and analysis of mean comparison were carried out using STATISTICA 3.1 (Dell Inc., Tulsa, OK, USA, StatSoft Polska, Kraków, Poland) software. The normality of the distribution was checked using Shapiro–Wilk, Lilliefors, and Kolmogorov–Smirnov tests. The means were compared using Tukey’s test at a significance level of *p* < 0.05.

### 2.5. Machine Learning Models for Distinguishing Fresh, Convective, and Infrared-Dried Date Fruit

The classification models were built based on selected image texture parameters of fresh and dried date fruit using WEKA 3.9 machine learning software (Machine Learning Group, University of Waikato, Hamilton, New Zealand) [21,22,23]. The image textures with the highest discriminative power were selected using the Best First and the correlation-based feature selection subset evaluator. The models for distinguishing fresh, convective, and infrared-dried date fruit were built using a 10-fold cross-validation mode by dividing the dataset into 10 parts and considering nine parts as the training sets and one part as the test set. This process was repeated 10 times for different training/test sets, and the result was the average of 10 estimates. The machine learning algorithms belonging to the groups of Bayes, Trees, Lazy, Functions, and Meta were tested to select algorithms that provided highly accurate results. The confusion matrices with accuracies for individual classes, average accuracies, and the values of True Positive Rate (TPR), False Positive Rate (FPR), Precision, Recall, F-Measure, Matthews Correlation Coefficient (MCC), Receiver Operating Characteristic Area (ROC Area), and Precision–Recall Area (PRC Area) (Equations (1)–(8)) [24,25,26,27] were determined.

(1) $$Accuracy=\frac{\left(TP+TN\right)}{TP+TN+FN+FP}$$

(2) $$TPR=Recall=\frac{TP}{TP+FN}$$

(3) $$FPR=\frac{FP}{FP+TN}$$

(4) $$Precision=\frac{TP}{TP+FP}$$

(5) $$F-Measure=\frac{2TP}{2TP+FP+FN}$$

(6) $$MCC=\frac{\left(TP*TN-FP*FN\right)}{\sqrt{\left(\left(TP+FP\right)\left(TP+FN\right)\left(TN+FP\right)\left(TN+FN\right)\right)}}$$

(7) ROC Area = Area Under TPR vs. FPR Curve

(8) PRC Area=Area Under Precision vs. Recall Curve

TP: True Positive; TN: True Negative; FP: False Positive; FN: False Negative

## 3. Results and Discussion

### 3.1. ‘Mejhoul’: Discrimination between Fresh, Convective, and Infrared Dried Fruit Based on Machine Learning Models and Selected Image Texture Parameters

The selected image texture parameters of fresh, convective-dried, and infrared-dried ‘Mejhoul’ date fruit samples were compared, and graphs of mean values are shown in Table 1. In the case of RHMean, when *R* was color channel *R* (red) from the RGB color space, and HMean was histogram’s mean, there was no significant difference (*p* > 0.05) between fresh and convective-dried fruit samples, and these samples were included in one homogenous group. For other analyzed image textures, such as GHMean, BHMean, LHMean, aHMean, bHMean, XHMean, YHMean, and ZHMean (the first letter means the color channel as follows: *G* (green) and *B* (blue) from the RGB color space; *L* (lightness component from black to white), *a* (red or green), and *b* (yellow or blue) from the Lab color space; and *X* (component with color information), *Y* (lightness), and *Z* (component with color information) from the XYZ color space, fresh, convective and infrared samples were significantly different.

Models developed based on selected texture parameters extracted from images in different color channels *R*, *G*, *B*, *L*, *a*, *b*, *X*, *Y*, *Z*, *U*, *V*, and *S* allowed for distinguishing fresh and dried ‘Mejhoul’ date fruit with very high correctness. The accuracy average of classification of fresh, convective-dried, and infrared-dried samples reached 99.33% for a model built using Random Forest (Table 2). However, slightly lower accuracies were obtained for models developed using IBk (99.25%), Multilayer Perceptron (98.67%), Logit Boost and Bayes Net (98.42%), and PART (97.83%). The higher differences occurred between fresh and infrared-dried ‘Mejhoul’ date fruit, whereas the greatest misclassification was observed between fresh and convective-dried fruit.

Other performance metrics, such as True Positive Rate (TPR), False Positive Rate (FPR), Precision, Recall, F-Measure, Matthews Correlation Coefficient (MCC), Receiver Operating Characteristic Area (ROC Area), and Precision–Recall Area (PRC Area) confirmed the highest correctness of the classification of infrared-dried ‘Mejhoul’ date fruit (Table 3). The greatest differentiation of infrared-dried fruit in terms of selected image textures from fresh and convective-dried samples was particularly visible in the case of the machine learning model built using Multilayer Perceptron. The values of TPR, Precision, Recall, F-Measure, MCC, ROC Area, and PRC Area were equal to 1.000, and FPR was 0.000. It confirmed that there was no mixing of cases between infrared-dried and other classes. In the case of each model, the lowest values of TPR, Recall, F-Measure, MCC, and ROC Area and the highest FPR were obtained for convective-dried ‘Mejhoul’ fruit. These results confirmed that convective-dried date fruit was classified with the lowest correctness.

### 3.2. ‘Boufeggous’: Discrimination between Fresh, Convective, and Infrared Dried Fruit Based on Machine Learning Models and Selected Texture Parameters

In the case of the ‘Boufeggous’ cultivar, the mean comparison of selected image texture parameters of fresh, convective-dried, and infrared-dried samples revealed the greatest similarity between fresh and convective-dried fruit. Among the nine image texture features analyzed for fresh and convective-dried ‘Boufeggous’ samples, only RHMean, GHMean, LHMean, and XHMean were in the same homogenous group with no statistically significant difference. Graphs of texture mean values are presented in Table 4.

For this date palm cultivar, classification accuracies were slightly lower (Table 5) than ‘Mejhoul’. The average accuracy of distinguishing fresh, convective-dried, and infrared-dried date fruit reached 94.33% for a model built using Random Forest, compared to 99.33% for ‘Mejhoul’. Nevertheless, the lowest average accuracy for analyzed models was observed for the IBk algorithm, and it was equal to 91.00%. 

As was reported for ‘Mejhoul’, fresh and infrared-dried ‘Boufeggous’ date fruit were distinguished with the highest correctness. Thus, the highest number of misclassified cases was between fresh and convective-dried fruit. In the case of the model developed using IBk, 57 cases belonging to the actual class of convective-dried ‘Boufeggous’ fruit were misclassified as fresh samples, and 47 cases of fresh fruit were incorrectly classified as convective-dried fruit.

The classification values of TPR, FPR, Precision, Recall, F-Measure, MCC, ROC Area, and PRC Area of fresh, convective-dried, and infrared-dried ‘Boufeggous’ date fruit are presented in Table 6. The highest classification accuracy of infrared-dried date fruit was confirmed by the lowest FPR and the highest values of other metrics. The value of 1.000 was obtained in the case of ROC Area and PRC Area for a model built using Random Forest and ROC Area for a model developed using Bayes Net. The lowest FPR was found for a model built by Bayes Net.

The above-mentioned tables showed an important trend in the variation in the above-mentioned texture parameters that can be ranged into two different groups: group with high values for ID drying (aHMean and bHMean); and group with lower values for ID drying (GHMean, BHMean, LHMean, XHMean, YHMean, and ZHMean). Nevertheless, both groups are characterized by convective drying texture features comprising fresh and infrared values. This trend may reflect significant texture variation between fresh, convective, and infrared dried ‘Mejhoul’ date fruit with high differences between ID and fresh compared to CD and fresh fruit. For ‘Boufeggous’, these findings are applied only for the case of aHMean and YHMean features. 

Regarding developed models, Random forest was the most accurate and predicting algorithm for both ‘Mejhoul’ and ‘Boufeggous’ while PART and lazy lBk were respectively the lower predicting models for ‘Mejhoul’ and ‘Boufeggous’. Such constatation was reported by [28] while comparing the effectiveness and accuracy of six algorithms used for orange (*Citrus sinensis*) classification. Among Logistic Regression, Naïve Bayes, Decision Trees, Neural Networks, K-nearest neighbors, and Random Forests models, authors found that Logistic Regression was more accurate and precise, with an accuracy of 91% and a precision score of 92%. 

In another study related to cultivar date fruit classification, [29] employed algorithms developed on the basis of ANN and LR approaches to discriminate between seven date fruit cultivars. The lowest accuracies of 91 and 92.2% compared to the results of this paper were obtained for distinguishing color, shape, and pomological features extracted from images.

In relation to these findings and the scope of this study, the previous literature data considered mainly the application of destructive analysis (instead of non-destructive methods) to assess date fruit quality in terms of physicochemical properties under different experimental conditions and various postharvest treatments [30,31,32,33]. In this way, mathematical models were used in addition to “response surface methodology” to express the drying kinetics and behavior of date fruit [34,35,36,37,38] without considering machine learning approaches for the specific case of date fruit drying. Nevertheless, machine learning models were employed for other fruit species to determine and assess dried fruit quality. For example, Raihen and Akter [39] developed deep-learning models for the classification of dried grapefruit types. Sağlam and Çetin [40] applied machine learning models to estimate the drying characteristics of dried apple slices by employing artificial neural networks, k-nearest neighbors, random forests, etc. For the three apple cultivars, the highest correlation coefficients were about 0.98 for moisture ratio estimation using a Random Forest algorithm. The effectiveness and usefulness of machine learning models in rapid estimation of chemical properties of apples were also proven with five machine learning algorithms [41]. Also, machine learning algorithms were used to predict the sweetness of dried bananas using Linear Discriminant Analysis (LDA), K Nearest Neighbors (KNN), Decision Tree (CART), Random Forest (RF), and Support Vector Machines (SVM) models. Among these five algorithms, authors reported that RF and SVM allowed for a high prediction accuracy of 86% [42]. The above-mentioned studies stated the importance of using ML algorithms to predict and estimate the biochemical properties of dried fruit with high accuracy, and the results presented in this paper are comparable in terms of high accuracy. Our study presented in this paper revealed the usefulness of machine learning models in determining the external quality of dried date fruit. However, further research can be related to the estimation of the physicochemical properties of date fruit using image processing and artificial intelligence.

## 4. Conclusions

Through this work, the effect of mild convective and infrared drying techniques on the changes in the external quality of date fruit was revealed in a non-destructive and objective manner using a flatbed scanner. The developed machine learning models, using selected texture parameters from images in different color channels *R*, *G*, *B*, *L*, *a*, *b*, *X*, *Y*, *Z*, *U*, *V*, and *S,* classified accurately fresh, convective-dried, and infrared-dried ‘Mejhoul’ and ‘Boufeggous’ date palm fruit. Thus, samples were classified with high correctness of 99.33% and 94.33 for ‘Mejhoul’ and ‘Boufeggous’, respectively, using the Random Forest algorithm, which was more accurate than other machine learning models tested under this work. Furthermore, the usefulness of image features and models built using artificial intelligence was confirmed and proved the greater effect of infrared drying on the improvement in the external appearance of date fruit for both cultivars.

From this perspective, selected texture features from date fruit images can be used as inputs to develop more accurate models using traditional machine learning and deep-learning algorithms that link those externally extracted features with other internal quality parameters such as biochemical and nutritional compounds. Additionally, other innovative drying methods can be evaluated for other date fruit cultivars with a focus on the elucidation of machine learning approaches that added value to the total quality assessment.

## Figures and Tables

**Table 1:** Mean values of selected image texture parameters of fresh, convective-dried, and infrared-dried ‘Mejhoul’ date fruit.

Image Texture Parameter	Fresh ‘Mejhoul’	Convective-Dried ‘Mejhoul’	Infrared-Dried ‘Mejhoul’
RHMean	70.35 a	70.08 ab	69.44 b
GHMean	59.65 a	57.44 b	55.54 c
BHMean	55.51 a	53.45 b	51.60 c
LHMean	91.94 a	90.28 b	88.77 c
aHMean	130.02 a	130.79 b	131.24 c
bHMean	130.46 a	130.73 b	130.91 b
XHMean	12.74 a	12.41 b	11.85 c
YHMean	12.58 a	12.08 b	11.40 c
ZHMean	10.56 a	10.07 b	9.30 c

Note. In the same row, means with different letters are significantly different (*p* < 0.05) according to Tukey HSD test.

**Table 2:** Distinguishing accuracies of fresh, convective, and infrared-dried ‘Mejhoul’ date fruit using machine learning models.

Algorithm	Predicted Class (Number of Cases)			Actual Class	Average Accuracy (%)
	Fresh ‘Mejhoul’	Convective-Dried ‘Mejhoul’	Infrared-Dried ‘Mejhoul’		
trees Random Forest	400	0	0	Fresh ‘Mejhoul’	99.33
	6	393	1	Convective-dried ‘Mejhoul’	
	0	1	399	Infrared-dried ‘Mejhoul’	
rules PART	399	1	0	Fresh ‘Mejhoul’	97.83
	23	376	1	Convective-dried ‘Mejhoul’	
	0	1	399	Infrared-dried ‘Mejhoul’	
meta Logit Boost	394	6	0	Fresh ‘Mejhoul’	98.42
	12	387	1	Convective-dried ‘Mejhoul’	
	0	0	400	Infrared-dried ‘Mejhoul’	
lazy IBk	400	0	0	Fresh ‘Mejhoul’	99.25
	8	391	1	Convective-dried ‘Mejhoul’	
	0	0	400	Infrared-dried ‘Mejhoul’	
functions Multilayer Perceptron	397	3	0	Fresh ‘Mejhoul’	98.67
	13	387	0	Convective-dried ‘Mejhoul’	
	0	0	400	Infrared-dried ‘Mejhoul’	
Bayes Bayes Net	391	9	0	Fresh ‘Mejhoul’	98.42
	9	390	1	Convective-dried ‘Mejhoul’	
	0	0	400	Infrared-dried ‘Mejhoul’	

**Table 3:** Performance metrics of distinguishing fresh and dried date fruit ‘Mejhoul’ using machine learning models developed based on texture parameters.

Algorithm	Class	TPR	FPR	Precision	Recall	F-Measure	MCC	ROC Area	PRC Area
trees Random Forest	Fresh ‘Mejhoul’	1.000	0.008	0.985	1.000	0.993	0.989	1.000	1.000
	Convective-dried ‘Mejhoul’	0.983	0.001	0.997	0.983	0.990	0.985	0.998	0.998
	Infrared-dried ‘Mejhoul’	0.998	0.001	0.998	0.998	0.998	0.996	1.000	1.000
rules PART	Fresh ‘Mejhoul’	0.998	0.029	0.945	0.998	0.971	0.956	0.985	0.939
	Convective-dried ‘Mejhoul’	0.940	0.003	0.995	0.940	0.967	0.951	0.966	0.970
	Infrared-dried ‘Mejhoul’	0.998	0.001	0.998	0.998	0.998	0.996	0.998	0.994
meta Logit Boost	Fresh ‘Mejhoul’	0.985	0.015	0.970	0.985	0.978	0.966	0.999	0.998
	Convective-dried ‘Mejhoul’	0.968	0.008	0.985	0.968	0.976	0.964	0.996	0.996
	Infrared-dried ‘Mejhoul’	1.000	0.001	0.998	1.000	0.999	0.998	0.999	0.998
lazy IBk	Fresh ‘Mejhoul’	1.000	0.010	0.980	1.000	0.990	0.985	0.991	0.966
	Convective-dried ‘Mejhoul’	0.978	0.000	1.000	0.978	0.989	0.983	0.981	0.985
	Infrared-dried ‘Mejhoul’	1.000	0.001	0.998	1.000	0.999	0.998	0.999	0.998
functions Multilayer Perceptron	Fresh ‘Mejhoul’	0.993	0.016	0.968	0.993	0.980	0.970	0.996	0.990
	Convective-dried ‘Mejhoul’	0.968	0.004	0.992	0.968	0.980	0.970	0.992	0.974
	Infrared-dried ‘Mejhoul’	1.000	0.000	1.000	1.000	1.000	1.000	1.000	1.000
Bayes Bayes Net	Fresh date fruit	0.978	0.011	0.978	0.978	0.978	0.966	0.999	0.998
	Convective-dried date fruit	0.975	0.011	0.977	0.975	0.976	0.964	0.997	0.996
	Infrared-dried date fruit	1.000	0.001	0.998	1.000	0.999	0.998	0.999	0.998

TPR—True Positive Rate; FPR—False Positive Rate; MCC—Matthews Correlation Coefficient; ROC Area—Receiver Operating Characteristic Area; PRC Area—Precision–Recall Area.

**Table 4:** Mean values of selected image texture parameters of fresh, convective-dried, and infrared-dried ‘Boufeggous’ date fruit.

Image Texture Parameter	Fresh ‘Boufeggous’	Convective-Dried ‘Boufeggous’	Infrared-Dried ‘Boufeggous’
RHMean	65.32 a	65.25 a	63.70 b
GHMean	56.54 a	56.18 a	53.20 b
BHMean	49.58 a	51.53 b	48.22 c
LHMean	88.10 a	88.04 a	85.40 b
aHMean	129.06 a	129.58 b	130.07 c
bHMean	131.64 a	130.59 b	131.01 c
XHMean	11.56 a	11.47 a	10.81 b
YHMean	11.61 a	11.60 a	10.74 b
ZHMean	9.08 a	9.72 b	8.84 c

Note. In the same row, means with different letters are significantly different (*p* < 0.05) according to Tukey HSD test.

**Table 5:** Distinguishing accuracies of fresh, convective, and infrared dried ‘Boufeggous’ date fruit using machine learning models.

Algorithm	Predicted Class (Number of Cases)			Actual Class	Average Accuracy (%)
	Fresh ‘Boufeggous’	Convective-Dried ‘Boufeggous’	Infrared-Dried ‘Boufeggous’		
trees Random Forest	377	22	1	Fresh ‘Boufeggous’	94.33
	42	356	2	Convective-dried ‘Boufeggous’	
	0	1	399	Infrared-dried ‘Boufeggous’	
rules PART	361	38	1	Fresh ‘Boufeggous’	92.25
	48	348	4	Convective-dried ‘Boufeggous’	
	0	2	398	Infrared-dried ‘Boufeggous’	
meta Logit Boost	367	31	2	Fresh ‘Boufeggous’	94.25
	35	365	0	Convective-dried ‘Boufeggous’	
	0	1	399	Infrared-dried ‘Boufeggous’	
lazy IBk	352	47	1	Fresh ‘Boufeggous’	91.00
	57	342	1	Convective-dried ‘Boufeggous’	
	1	1	398	Infrared-dried ‘Boufeggous’	
functions Multilayer Perceptron	370	26	4	Fresh ‘Boufeggous’	93.92
	41	359	0	Convective-dried ‘Boufeggous’	
	1	1	398	Infrared-dried ‘Boufeggous’	
Bayes Bayes Net	373	26	1	Fresh ‘Boufeggous’	93.67
	43	357	0	Convective-dried ‘Boufeggous’	
	1	5	394	Infrared-dried ‘Boufeggous’	

**Table 6:** Performance metrics of distinguishing fresh and dried ‘Boufeggous’ date fruit using machine learning models developed based on texture parameters.

Algorithm	Class	TPR	FPR	Precision	Recall	F-Measure	MCC	ROC Area	PRC Area
trees Random Forest	Fresh ‘Boufeggous’	0.943	0.053	0.900	0.943	0.921	0.880	0.990	0.980
	Convective-dried ‘Boufeggous’	0.890	0.029	0.939	0.890	0.914	0.873	0.990	0.980
	Infrared-dried ‘Boufeggous’	0.998	0.004	0.993	0.998	0.995	0.993	1.000	1.000
rules PART	Fresh ‘Boufeggous’	0.903	0.060	0.883	0.903	0.892	0.838	0.961	0.938
	Convective-dried ‘Boufeggous’	0.870	0.050	0.897	0.870	0.883	0.826	0.967	0.927
	Infrared-dried ‘Boufeggous’	0.995	0.006	0.988	0.995	0.991	0.987	0.995	0.984
meta Logit Boost	Fresh ‘Boufeggous’	0.918	0.044	0.913	0.918	0.915	0.873	0.988	0.975
	Convective-dried ‘Boufeggous’	0.913	0.040	0.919	0.913	0.916	0.874	0.987	0.978
	Infrared-dried ‘Boufeggous’	0.998	0.003	0.995	0.998	0.996	0.994	0.999	0.999
lazy IBk	Fresh ‘Boufeggous’	0.880	0.073	0.859	0.880	0.869	0.803	0.905	0.800
	Convective-dried ‘Boufeggous’	0.855	0.060	0.877	0.855	0.866	0.800	0.899	0.804
	Infrared-dried ‘Boufeggous’	0.995	0.003	0.995	0.995	0.995	0.993	0.996	0.992
functions Multilayer Perceptron	Fresh ‘Boufeggous’	0.925	0.053	0.898	0.925	0.911	0.866	0.973	0.948
	Convective-dried ‘Boufeggous’	0.898	0.034	0.930	0.898	0.913	0.872	0.979	0.963
	Infrared-dried ‘Boufeggous’	0.995	0.005	0.990	0.995	0.993	0.989	0.998	0.995
Bayes Bayes Net	Fresh ‘Boufeggous’	0.933	0.055	0.894	0.933	0.913	0.869	0.982	0.964
	Convective-dried ‘Boufeggous’	0.893	0.039	0.920	0.893	0.906	0.860	0.981	0.966
	Infrared-dried ‘Boufeggous’	0.985	0.001	0.997	0.985	0.991	0.987	1.000	0.999

TPR—True Positive Rate; FPR—False Positive Rate; MCC—Matthews Correlation Coefficient; ROC Area—Receiver Operating Characteristic Area; PRC Area—Precision–Recall Area.

## Data Availability

The original contributions presented in the study are included in the article, further inquiries can be directed to the corresponding author.
